# Decolorization of recalcitrant dyes by a multicopper oxidase produced by *Iodidimonas* sp. Q-1 with iodide as a novel inorganic natural redox mediator

**DOI:** 10.1038/s41598-018-25043-1

**Published:** 2018-04-30

**Authors:** Taro Taguchi, Kyota Ebihara, Chihiro Yanagisaki, Jun Yoshikawa, Hirofumi Horiguchi, Seigo Amachi

**Affiliations:** 1 Graduate School of Horticulture, Chiba University, 648 Matsudo, Matsudo City, Chiba 271-8510 Japan; 2 GODO SHUSEI Co. Ltd., 250 Nakahara, Kamihongo, Matsudo City, Chiba 271-0064 Japan

## Abstract

A multicopper oxidase (IOX) produced by *Iodidimonas* sp. Q-1 has high catalytic efficiency for iodide (I^−^) oxidation to form molecular iodine (I_2_). In this study, the potential capacity of IOX for decolorization of recalcitrant dyes was determined. Although IOX did not decolorize any dyes in the absence of redox mediator, significant decolorization of Orange G, Indigo Carmine, Amido Black, and Remazol Brilliant Blue R (RBBR) was observed in the presence of iodide. Addition of 0.1 mM iodide was sufficient to decolorize a total of 3 mM Indigo Carmine, suggesting that iodide functions as a mediator. Such mediator-like function of iodide was not observed in commercially available fungal laccases. The IOX-iodide decolorization system showed much alkaline pH optima of 5.5–6.5 and stronger salt tolerance than fungal laccases did. In addition, actual wastewater discharged from a dyeing factory could be decolorized more than 50% by the system. Since iodide is naturally occurring, non-toxic, and cheaper than common synthetic mediators, the IOX-iodide system is potentially more advantageous than fungal laccase-mediator systems for decolorization of recalcitrant dyes.

## Introduction

*Iodidimonas* sp. Q-1 is a heterotrophic iodide-oxidizing bacterium isolated from iodide-rich brine water in Japan, and oxidizes iodide (I^−^) to form molecular iodine (I_2_)^1,2^. Recently, iodide-oxidizing enzyme (IOX) of strain Q-1 was purified and characterized^3^. IOX is an extracellular oxidase and the addition of Cu^2+^ ions to the culture medium significantly enhanced enzyme production by this strain. Purified IOX exhibited significant activity towards not only iodide but also phenolic compounds such as 2,2′-azino-bis(3-ethylbenzothiazoline-6-sulfonate) (ABTS), syringaldazine, 2,6-dimethoxyphenol, *p*-phenylenediamine, and hydroquinone. A comparison of several internal amino acid sequences with a draft genome sequence of strain Q-1^4^ revealed that at least two proteins, IoxA and IoxC, are involved in iodide oxidation. Among these, IoxA was a putative multicopper oxidase (MCO) with four conserved copper-binding regions, but it was phylogenetically distinct from other known bacterial MCOs such as CueO, CumA, CopA, and CotA^3^. MCOs are a family of copper-containing enzymes comprising laccases, ascorbate oxidases, ferroxidases, and ceruloplasmin, and couple the oxidation of a wide variety of substrates with a four-electron reduction of molecular oxygen to form water^5,6^. Enhancement of enzyme activity by Cu^2+^ ion has been reported in CueO^7^ and YacK^8^ of *E*. *coli*, and Mn^2+^ oxidases of *Pseudomonas putida* GB-1^9^ and *Bacillus* sp. SG-1^10^.

One distinctive feature of IOX that differs from other MCOs is its high catalytic efficiency (*k*_cat_/*K*_*m*_) for iodide. Suzuki *et al*.^3^ found that the catalytic efficiency of IOX for iodide was 2**–**5 orders higher than that for the fungal laccases. Based on this unique characteristic and on the fact that molecular iodine has a broad antimicrobial spectrum against a wide variety of microorganisms, Yuliana *et al*.^11^ recently prepared a novel enzyme-based antimicrobial system consisting of IOX and iodide, and determined its antimicrobial activity. Both Gram-positive and Gram-negative bacteria tested were killed completely within 5 min by the IOX-iodide system. Furthermore, sporicidal activity of the IOX-iodide system against *Bacillus* and *Geobacillus* spores was much stronger than that of a common iodophor, povidone iodine.

More than 10,000 commercial dyes exist today, whose production accounts for 0.8 million tons per year, and at least 10% of the used dyestuff is released into the environment through wastewater^12,13^. Most of the synthetic dyes are recalcitrant, and resistant to light, water, temperature, and microbial attack. In addition, wastewaters discharged by dyeing industries are toxic to animals and plants, since many dyes are made from known carcinogens^14,15^. To remove synthetic dyes from industrial effluents, various physicochemical methods such as adsorption on sorbents, oxidation by chemicals and photo-degradation, filtration, and ion exchange are used^16^. However, these methods are sometimes uneconomical, and generate by-products or concentrated sludge. Thus, biological degradation and decolorization of synthetic dyes by microorganisms or by their enzymes has recently been an area of intense research as an alternative cost-effective and eco-friendly method^17,18^. Generally, laccases can catalyze the oxidation of various phenolic compounds. Owing to their broad substrate range, laccases have been extensively studied for various industrial and biotechnological applications, including fabric bleaching, cork modification, paper pulp delignification, and dye decolorization^19–21^. The substrate range of certain laccases is dramatically increased in the presence of redox mediators such as ABTS and 1-hydroxybenzotriazole (HOBt).

To date, most of the known laccases have fungal or plant origins. However, many laccase-like MCOs have been discovered in bacteria and bacterial genomes in the last two decades^6^. Although only fungal laccases are currently used in industrial processes, bacterial MCOs possess advantages over fungal MCOs such as their wider range of pH optima, higher stability against temperature and salt, higher yield of enzyme production, and easier overexpression using genetic engineering techniques^22^. In this study, we determined the potential decolorization activity of IOX towards various synthetic dyes in the absence and presence of redox mediators. Special attention was paid to understand whether iodide could function as a redox mediator for IOX, since a good laccase substrate can be an ideal redox mediator^19^. Decolorization of actual wastewater discharged from a dyeing factory was also tested. The decolorization activity of IOX was routinely compared to that of commercially available fungal laccases, *i*.*e*. *Trametes versicolor* laccase (TvL) and *Pleurotus ostreatus* laccase (PoL).

## Results

### Decolorization of Orange G by IOX and fungal laccases

**Figure 1:**
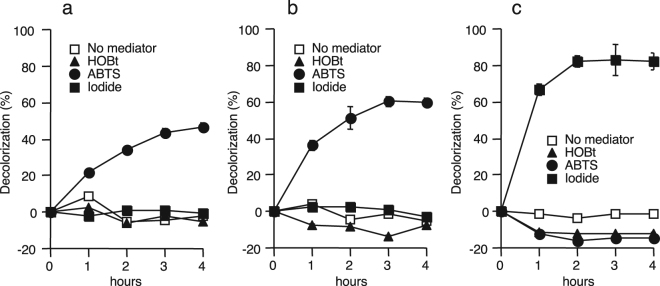
Decolorization of Orange G by TvL (**a**), PoL (**b**), and IOX (**c**) in the absence and presence of redox mediators. The reaction mixture contained 10 mU mL^−1^ of enzyme, 0.3 mM Orange G, and 20 mM sodium acetate buffer (pH 5.0 for fungal laccases and pH 5.5 for IOX). Redox mediators used were 0.1 mM of ABTS, HOBt, and potassium iodide. Symbols represent the mean values obtained for triplicate determinations, and bars indicate standard deviations. In most cases, standard deviation values are smaller than those denoted by the symbols.

A 10 mU mL^−1^ each of IOX and fungal laccases was incubated with 0.3 mM Orange G, an azo dye, for 4 h in the presence or absence of potential redox mediators. As shown in Fig. 1a, Orange G decolorization by TvL was observed in the presence of 100 µM ABTS, while no significant decolorization occurred in the absence of mediator or in the presence of HOBt and iodide. After 4 h, decolorization efficiencies in the presence of ABTS was 47%. Significant decolorization of Orange G by PoL (60% at 4 h) was also observed only in the presence of ABTS (Fig. 1b). In the case of IOX, no significant decolorization occurred in the presence of ABTS (Fig. 1c). However, 82% decolorization was observed in the presence of iodide. No decolorization occurred in the absence of IOX, indicating that the decolorization was an enzymatic process (Fig. S1). Such enhancement of decolorization by iodide was not observed in TvL and PoL (Fig. 1a and b).

### Decolorization of various dyes by IOX

Decolorization of an indigoid dye (Indigo Carmine), other azo dye (Amido Black), and an anthraquinone dye (RBBR) by IOX was determined. As shown in Supplemental Fig. S1, IOX did not decolorize these dyes significantly in the absence of iodide. However, in the presence of iodide, the color of Indigo Carmine, Amido Black, and RBBR changed to light yellow, light pink, and light green, respectively.

### UV–VIS spectrophotometric analysis

The spectrophotometric analysis of Orange G, Amido Black, and RBBR before and after decolorization revealed that the absorbance at 200–800 nm significantly changed with time (Supplemental Fig. S2). In all cases, the absorbance peaks in the visible region, *i*.*e*. the maximum visible absorptions at 480, 620, and 595 nm for Orange G, Amido Black, and RBBR, respectively, decreased with time. This was probably due to the breakdown of the chromophoric group in the dyes.

### Effect of iodide on dye decolorization

**Figure 2:**
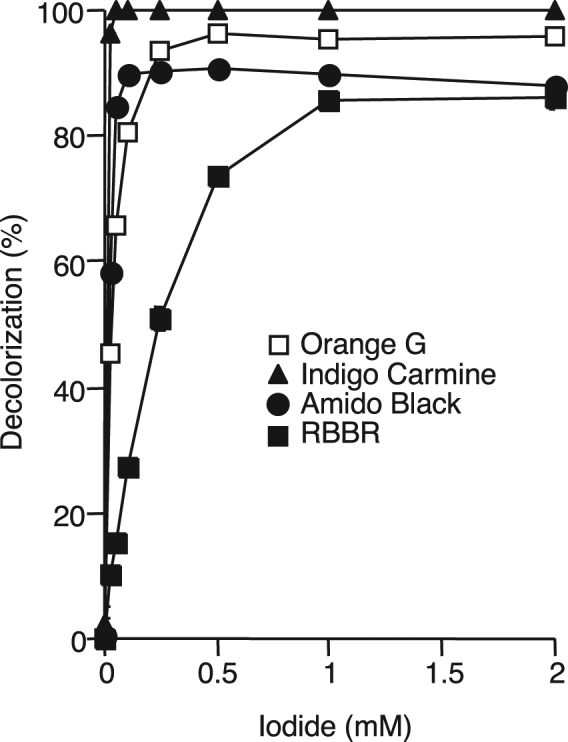
Effect of iodide concentration on decolorization of various dyes by IOX. The reaction mixture contained 10 mU mL^−1^ of IOX, 0.3 mM each of dye, 0.025–2 mM potassium iodide, and 20 mM sodium acetate buffer (pH 5.5). The reaction mixture was incubated for 2 h (Indigo Carmine), 4 h (Amido Black), or 8 h (Orange G and RBBR).

Effect of iodide concentration on decolorization of 0.3 mM each of Orange G, Indigo Carmine, Amido Black, and RBBR by IOX was determined. As shown in Fig. 2, 0.1 mM iodide was sufficient for 71–99% decolorization of the azo and indigoid dyes, while 1 mM iodide was required for 78% decolorization of RBBR.

### Successive decolorization of Indigo Carmine

**Figure 3:**
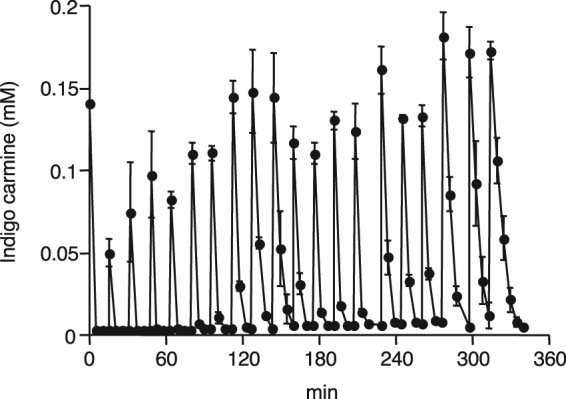
Successive decolorization of Indigo Carmine by IOX. The reaction mixture contained 10 mU mL^−1^ of IOX, 0.15 mM Indigo Carmine, 0.1 mM iodide, and 20 mM sodium acetate buffer (pH 5.5). After complete decolorization, 0.15 mM of Indigo Carmine was sequentially added to the reaction mixture a total of 20 times. The molar absorption coefficients of Indigo Carmine were determined in 20 mM sodium acetate buffer (pH 5.5), and 13.3 mM^−1^·cm^−1^ was used for quantification.

In order to determine the persistence of the IOX-iodide decolorization system, 0.15 mM of Indigo Carmine was sequentially added each time to the reaction mixture a total of 20 times in the presence of IOX and 0.1 mM iodide. As shown in Fig. 3, the rate of decolorization did not decrease until the 14^th^ addition of Indigo Carmine. Following this, the rate decreased gradually.

### Effect of pH on decolorization

**Figure 4:**
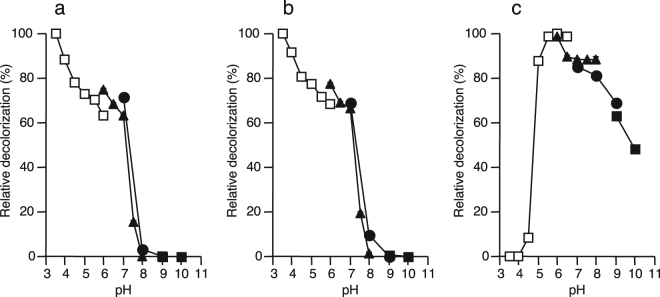
Effect of pH on Orange G decolorization activities by TvL (**a**), PoL (**b**), and IOX (**c**). The reaction mixture contained 10 mU mL^−1^ each of enzyme, 0.3 mM of dye, and 20 mM of appropriate buffers. Potassium iodide (for IOX) or ABTS (for TvL and PoL) was also added at 0.1 mM. Buffers used were sodium acetate (open squares), potassium phosphate (triangles), Tris-HCl (circles), and glycine-NaOH (closed squares). The reaction time was 48 h. Percentages decolorization at relative decolorization of 100% by TvL, PoL, and IOX were 86%, 93%, and 80%, respectively.

The effect of pH on decolorization of Orange G was determined with TvL, PoL, and IOX (Fig. 4). Both TvL and PoL showed acidic pH optima of 3.5, where decolorization efficiency reached 86–93% (Fig. 4a and b). In contrast, IOX showed nearly neutral pH optima of 5.5–6.5, where decolorization efficiency of 79–80% was obtained (Fig. 4c). While both TvL and PoL did not decolorize Orange G at pH of more than 8.0, IOX still maintained 49–81% of relative recolorization activity at these pH regions. In contrast, IOX could not decolorize Orange G at pH of less than 4.0.

### Effect of NaCl on decolorization

**Figure 5:**
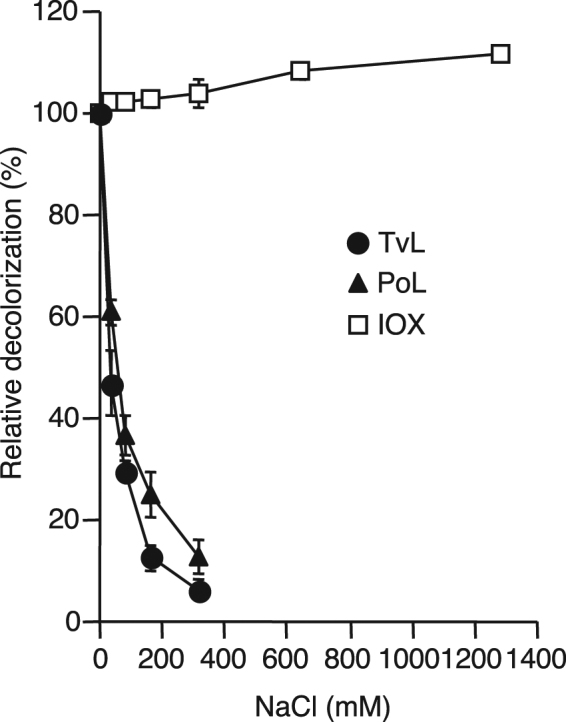
Effect of sodium chloride concentration on Orange G decolorization activity by TvL, PoL, and IOX. The reaction condition was same as described in the legend of Fig. 5. The reaction time was 2 h. Percentages decolorization at relative decolorization of 100% by TvL, PoL, and IOX were 64%, 75%, and 87%, respectively.

The effect of NaCl concentration on the decolorization of Orange G was determined (Fig. 5). Relative decolorization activities of both TvL and PoL decreased with the increase in NaCl concentration. At 320 mM NaCl, the relative decolorization activities of TvL and PoL were only 6.3% and 13%, respectively. In contrast, decolorization activity of IOX was not inhibited significantly by NaCl, and still maintained 100% relative activity at 1,280 mM NaCl.

### Effect of temperature on decolorization

The temperature stability of the enzymatic decolorization of Orange G was determined after 30-min treatment of IOX and fungal laccases at various temperatures. As shown in Supplemental Fig. S3, all enzymes maintained nearly 90% relative decolorization activities after the 60 °C-treatment. However, they showed only 9.5 to 14% relative decolorization activities after the 70 °C-treatment. After the treatment at 80 °C, all enzymes showed relative decolorization activities of less than 4.0%.

### Decolorization of industrial wastewater

**Figure 6:**
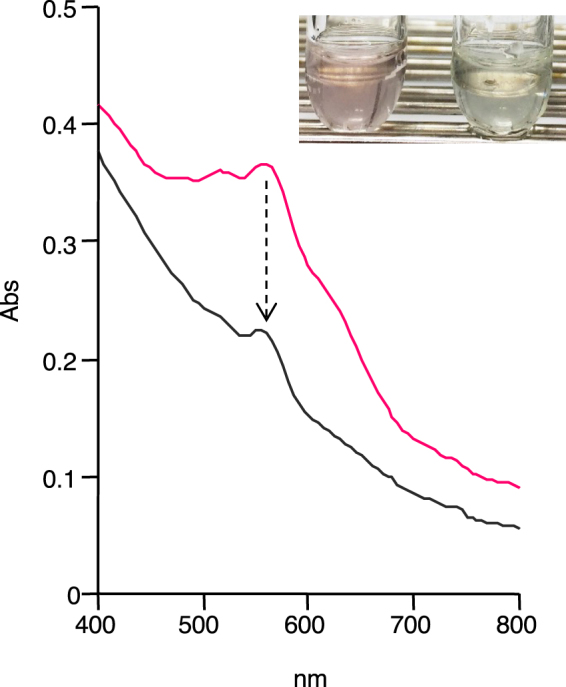
Decolorization of wastewater collected from a dyeing factory by IOX. The reaction mixture (1.5 mL) contained the wastewater (1.3 mL), 100 mU mL^−1^ of IOX, and 1 mM iodide. The reaction time was 2 h. UV-visible absorbance spectra before (pink line) and after (black line) the decolorization are shown. Inlet shows a photograph taken before (left) and after (right) the decolorization.

Actual wastewater (pH8.8) containing reactive dyes was collected from a dyeing factory, and used for decolorization experiment with IOX. In this experiment, iodide concentration was increased to 1 mM, since 0.1 mM iodide did not show significant mediator-like function in the wastewater decolorization. As shown in Fig. 6, IOX achieved 46% decolorization of wastewater within 2 h in the presence of iodide. Decolorization reaction almost ended within 1 h, and much more decolorization did not proceed even after much longer incubation time. When pH of the wastewater was adjusted to 5.0, 7.0, and 9.5, 62%, 48%, and 50% decolorization was observed within 2 h, respectively.

## Discussion

Since Wong and Yu^23^ first reported decolorization of synthetic dyes by TvL, a large number of white rot fungi and their laccases have been studied for their capacity for decolorization^17^. It is well-known that redox mediators such as ABTS, HOBt, and some lignin-derived compounds sometimes enhance enzyme action dramatically^19^. However, significantly fewer efforts have been made to understand the potential decolorization activity of bacterial laccase-like MCOs, except for a spore coat laccase-like protein (CotA) of *Bacillus* spp^24–27^. In this study, we found that IOX, a novel MCO produced by an iodide-oxidizing bacterium *Iodidimonas* sp. Q-1^3^, is able to decolorize various synthetic dyes in the presence of iodide. To the best of our knowledge, such mediator-like function of iodide has not been reported thus far in enzymatic decolorization of synthetic dyes.

Generally, iodide is a very poor substrate for MCOs, including fungal laccases. We previously reported that the catalytic efficiency of IOX for iodide (9.41 × 10^5^) was 3–5 orders higher than that for *Trametes multicolor* and *Myceliophthora thermophila* laccases^3^. Considering that an ideal redox mediator must be a good laccase substrate^19^, it is not surprising if iodide serves as a redox mediator for IOX. In the decolorization of Orange G, Amido Black and Indigo Carmine, only 0.1 mM iodide was sufficient for 80–100% decolorization, while initial concentration of these dyes was 0.3 mM (Fig. 2). In addition, complete decolorization was still observed even after the sequential addition of 0.15 mM Indigo Carmine to the reaction mixture for a total of 20 times (Fig. 3). This indicates that 0.1 mM iodide is sufficient for decolorization of 3 mM Indigo Carmine. Therefore, our results strongly suggest that molecular iodine (I_2_) is not simply added to the synthetic dyes to form iodinated compounds, but is reduced again to iodide by the dyes and repeats the redox cycling as a mediator. Although the redox potential of the iodide/iodine couple is relatively low (+0.536 V versus the standard hydrogen electrode) compared with that of ABTS (+0.670 to +1.08 V), this could increase if certain unstable radicals such as atomic iodine (I˙) and diiodide (I_2_^−^˙) are involved in the reaction^28^. Further study is needed to fully understand the iodine species actually serving as a redox mediator in the IOX-catalyzed decolorization. In addition, it is also important to identify the chemical structure of synthetic dyes after the decolorization.

It is noteworthy that the IOX-catalyzed decolorization had much alkaline pH optima compared with the fungal laccase (Fig. 4). Usually, fungal laccases exhibit pH optima in the acidic pH^21^. For example, the optimal pH of RBBR decolorization by *Funalia trogii* and *Ganoderma lucidum* laccases were reported to be pH 3 and pH 4, respectively^29,30^. Since the activity of IOX itself is relatively stable under alkaline conditions^3^, it is possible that its decolorization activity is also stable at high pH range. Certain dyes such as indigo dyes are used under alkaline conditions. Thus, IOX can be used for decolorization of such dyes without pH adjustment of the wastewater. IOX also showed strong salt tolerance (Fig. 5). This unique feature of the IOX-catalyzed decolorization may reflect the original brine environment from which *Iodidimonas* sp. Q-1 was isolated^1^. In addition to 0.6–1.2 mM of iodide, brine waters in Japan usually contain approximately 500 mM chloride, and have pH values of 7.7–8.0^31^. The efficient decolorization activity of IOX under high salts concentrations makes it a promising enzyme for application to the degradation of textile effluents, since effluents from textile and pulp industries usually contain high levels of salts^32^.

Claus *et al*.^33^ first found that dye decolorization by laccase was enhanced in the presence of a redox mediator. Since then, a wide variety of synthetic mediators such as ABTS, HOBt, violuric acid, and *N*-hydroxyacetanilide have been tested for laccase-catalyzed decolorization. However, these synthetic mediators are expensive and possibly generate toxic species, which hinder the practical use of the laccase-mediator system. Thus, lignin-derived compounds such as acetosyringone, syringaldehyde, and *p*-hydroxycinnamic acids are expected as environmentally friendly natural mediators^34,35^. Iodide, a reduced form of iodine, is distributed widely in the environment and has no known toxicity. Even in the oxidized form, molecular iodine has been used for wound care for more than 150 years as Lugol’s solution, and more recently as iodophores^36^. Furthermore, iodide is very cheap compared with not only synthetic mediators but also natural mediators. Although the exact redox mechanism of the IOX-catalyzed dye decolorization system is still unclear, various advantages of iodide over known mediators make this system a suitable candidate for a wide range of industrial applications.

## Methods

### Microorganism, culture condition and preparation of enzymes

*Iodidimonas* sp. Q-1 (JCM17846), previously isolated from iodide-rich natural gas brine in Miyazaki prefecture, Japan^1^, was grown aerobically at 30 °C in Marine Broth 2216 (Becton Dickinson, Sparks, MD, USA) supplemented with 40 µM CuCl_2_·2H_2_O.

For preparation of IOX, culture broth of strain Q-1 grown for 48 h was centrifuged at 6,000 × *g* for 10 min at 4 °C, and the supernatant was used directly as a crude IOX solution. Our previous study showed that almost all protein included in the supernatant was IOX^3^. Although the crude IOX solution included various salts originating from Marine Broth 2216, they were diluted approximately 700 times in the reaction mixture for decolorization (see below). Thus, additional dialysis of the crude IOX solution was not performed in this study. The crude IOX solution was stored at −30 °C until used. The enzyme activity of IOX was routinely assayed. The reaction mixture contained IOX, 10 mM potassium iodide, and 20 mM sodium acetate buffer (pH 5.5). The reaction was initiated by addition of IOX at 30 °C. The oxidation of iodide was measured spectrophotometrically by the absorbance increase at 353 nm using ε = 25.5 mM^−1^·cm^−1^. One unit of enzyme activity was defined as the amount of enzyme catalyzing the oxidation of 1 µmol of iodide per min at 30 °C. The enzyme activity of crude IOX solution was approximately 7,000 mU mL^−1^.

Laccases from TvL and PoL were purchased from Sigma-Aldrich (St. Louis, MO, USA). The enzyme activity of fungal laccases was assayed in the reaction mixture containing TvL or PoL, 0.1 mM ABTS, and 20 mM sodium acetate buffer (pH 5.0) at 30 °C. The oxidation of ABTS was measured at 420 nm using ε = 36.0 mM^−1^·cm^−1^.

### Decolorization experiments

The reaction mixture (1.5 mL) for decolorization of Orange G (Acid Orange 10; Nacalai Tesque, Kyoto, Japan) contained 10 mU mL^−1^ of IOX or fungal laccases (TvL or PoL), 0.3 mM dye, 100 µM each of ABTS, HOBt, or iodide (KI) as a potential redox mediator, and 20 mM sodium acetate buffer (pH 5.5 for IOX or 5.0 for fungal laccases). The reaction mixture was incubated at 30 °C, and absorbance at 480 nm was periodically determined for the calculation of decolorization efficiency according to the following formula: decolorization efficiency (%) = (*A*_1_ − *A*_2_)/*A*_1_ × 100, where *A*_1_ and *A*_2_ represent the absorbance at time zero and at the sampling time, respectively.

For decolorization of other dyes, Indigo Carmine (Wako Pure Chemical Industries), Amido Black (Acid Black 10B; Wako Pure Chemical Industries), and Remazol Brilliant Blue R (Reactive Blue 19, RBBR, Sigma-Aldrich) were added to the reaction mixture at a final concentration of 0.3 mM, and decolorization efficiency was determined at 600, 525, and 595 nm, respectively. When successive decolorization by IOX was observed, 0.15 mM of Indigo Carmine was sequentially added each time to the reaction mixture a total of 20 times.

### Effect of iodide, pH, sodium chloride, and temperature on decolorization

The effect of iodide concentration on dye decolorization was determined in the presence of 0.025–2 mM iodide under the assay conditions described above. The effect of pH on decolorization was examined with 20 mM sodium acetate buffer (pH 3.5–6.0), 20 mM potassium phosphate buffer (pH 6.0–8.0), 20 mM Tris-HCl buffer (pH 7.0–9.0), and 20 mM glycine-NaOH buffer (pH 9.0–10.0). The effect of NaCl was determined in the presence of 0–1,280 mM NaCl under the assay conditions described above. The temperature stability of the enzymatic decolorization was measured after 30-min treatment of the enzymes at various temperatures.

### Decolorization of industrial wastewater

Actual wastewater was collected from a dyeing factory located at Matsudo city (Kikawa Co., Ltd.). The wastewater contained both reactive dyes and pigments. Before use, the wastewater was centrifuged at 10,000 × *g* for 10 min at 4 °C to remove suspended matter. After the centrifugation, the supernatant (pH 8.8) showed the maximum absorbance at 590 nm. The reaction mixture (1.5 mL) contained the supernatant (1.3 mL), 100 mU mL^−1^ of IOX, and 1 mM iodide. In some cases, pH of the wastewater was adjusted to 5.0, 7.0, or 9.5 with HCl or NaOH.

### UV–visible absorbance spectra

Absorption spectra of Orange G, Amido Black, RBBR, and industrial wastewater before and after decolorization, were scanned between 200–800 nm by a BioSpec-nano spectrophotometer (Shimadzu, Kyoto, Japan).

### Data availability

The datasets generated during the current study are available from the corresponding author on reasonable request.

## Electronic supplementary material

Supplemental figures
