# Supplementary material for: Decolorization of recalcitrant dyes by a multicopper oxidase produced by Iodidimonas sp. Q-1 with iodide as a novel inorganic natural redox mediator
Source: Sci Rep. 2018 Apr 30;8:6717. doi: 10.1038/s41598-018-25043-1 (PMC5928188; doi:10.1038/s41598-018-25043-1)
Supplement: Supplementary file 1 — Supplemental figures [file 41598_2018_25043_MOESM1_ESM.pdf]

Supplemental information

Decolorization of recalcitrant dyes by a multicopper oxidase produced by *Iodidimonas* sp. Q-1 with iodide as a novel inorganic natural redox mediator

Taro Taguchi [1], Kyota Ebihara [1], Chihiro Yanagisaki [1], Jun Yoshikawa [2], Hirofumi Horiguchi [2] & Seigo Amachi [1,*]

[1]Graduate School of Horticulture, Chiba University, 648 Matsudo, Matsudo City, Chiba 271-8510, Japan. [2]GODO SHUSEI Co. Ltd., 250 Nakahara, Kamihongo, Matsudo City, Chiba 271-0064, Japan. Correspondence and requests for materials should be addressed to S. A. (email: amachi@faculty.chiba-u.jp)

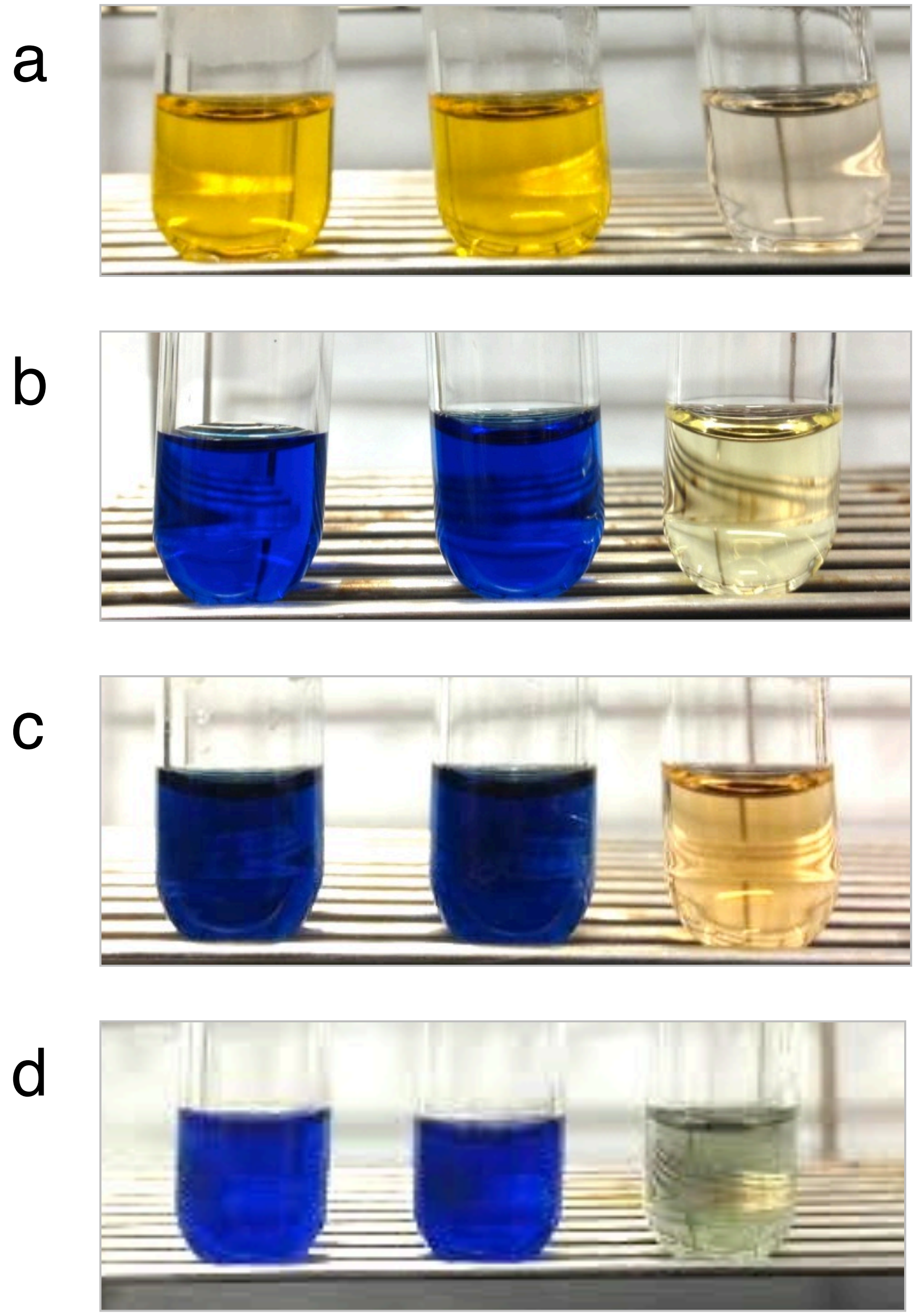

Supplemental Figure S1. Photographs taken after the decolorization of Orange G (a), Indigo Carmine (b), Amido Black (c), and RBBR (d) by the IOX-iodide system. The reaction time was 5 h. Left and middle tubes indicate negative controls in which the dyes were incubated without IOX and without potassium iodide, respectively. In right tubes, the dyes were incubated with both IOX and potassium iodide.

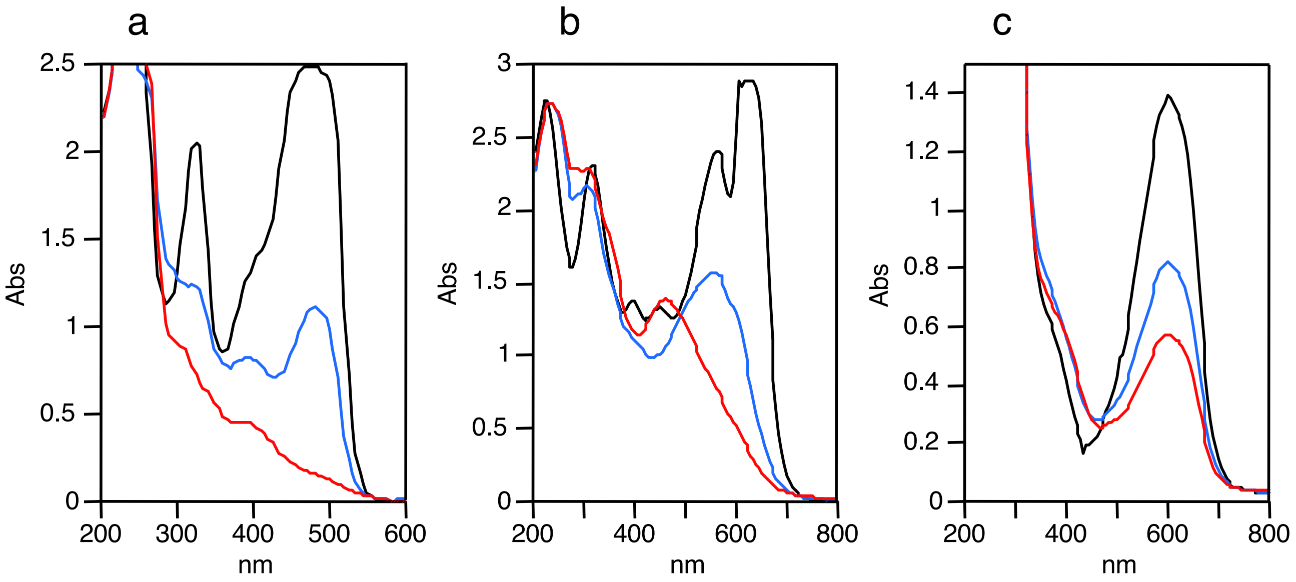

Supplemental Figure S2. UV-visible absorbance spectra of Orange G (a), Amido Black (b), and RBBR (c) before and after decolorization. The reaction mixture contained 10 mU mL^{-1} of IOX, 0.1 (Orange G) or 0.3 mM (RBBR and Amido Black) of dye, 0.1 mM potassium iodide, and 20 mM sodium acetate buffer (pH 5.5). (a) The reaction mixture was scanned at 0 (black line), 5 (blue line), and 10 min (red line). (b) The reaction mixture was scanned at 0 (black line), 30 (blue line), and 120 min (red line). (c) The reaction mixture was scanned at 0 (black line), 120 (blue line), and 240 min (red line).

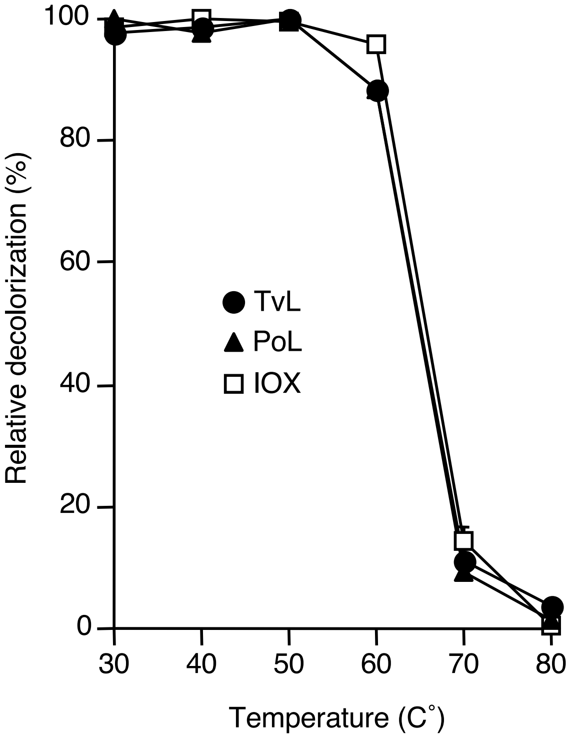

Supplemental Figure S3. The temperature stability of Orange G decolorization activity of TvL, PoL, and IOX. The reaction condition was same as described in the legend of Fig. 5, but the enzymes were exposed at various temperatures for 30 min before the decolorization reaction. The reaction time was 4 h.
